# Supplementary material for: Improving the immunogenicity of E. coli FimH via multivalent display on I53-50 nanoparticles
Source: Front Immunol. 2026 Jul 21;17:1872554. doi: 10.3389/fimmu.2026.1872554 (PMC13433762; doi:10.3389/fimmu.2026.1872554)
Supplement: Supplementary file 1 [file DataSheet1.pdf]

Supplementary Materials

Table S1. Amino acid sequences of FimH-I53-50A and I53-50B components

**Construct**	**Amino acid sequence***
FimH$_{LD}$-I53-50A	METDTLLLWVLLLWVPGSTGFACKTASGTAIPIGGGSANVYVNLAPAVNVGQNLVVDLSTQIFCHNDYPETITDYVTLQRGSAYGGVLSSFSGTVKYSGSSYPFPTTSETPRVVYNSRTDKPWPVALYLTPVSSAGGVAIKAGSLIAVLILRQTNNYNSDDFQFVWNIYANNDVVVPTGGGSGGSGSGEKAAKAEEAARKIEELFKKHKIVAVLRANSVEEAIEKAVAVFAGGVHLIEITFTVPDADTVIKALSVLKEKGAIIGAGTVTSVEQLRKAVESGAEFIVSPHLDEEISQFLKEKGVFYMPGVMTPTELVKAMKLGHTILKLFPGEVVGPQFVKAMKGPFPNVKFVPTGGVNLDNVLEWFKAGVLAVGVGSALVKGTPDEVREKAKAFVEKIRGLTEGSLEHHHHHH
FimH-DSG-I53-50A	METDTLLLWVLLLWVPGSTGFACKTASGTAIPIGAASANVYVNLAPAVNVGQNLVVDLSTQIFCHNDYPETITDYVTLQRGSAYGGVLSSFSGTVKYSGSSYPFPTTSETPRVVYNSRTDKPWPVALYLTPVSSAGGVAIKAGSLIAVLILRQTNNYNSDDFQFVWNIYANNDVVVPTGGCDVSARDVTVTLPDYPGSVPIPLTVYCAKSQNLGYYLSGTTADAGNSIFTNTASFSPAQGVGVQLTRSGTIIPASNTVSLGAVGTSAVSLGLTANYARTGGQVTAGNVQSIIGVTFVYQGGSSGGGADVTITVNGKVVAKGSGGSGSGEKAAKAEEAARKIEELFKKHKIVAVLRANSVEEAIEKAVAVFAGGVHLIEITFTVPDADTVIKALSVLKEKGAIIGAGTVTSVEQLRKAVESGAEFIVSPHLDEEISQFLKEKGVFYMPGVMTPTELVKAMKLGHTILKLFPGEVVGPQFVKAMKGPFPNVKFVPTGGVNLDNVLEWFKAGVLAVGVGSALVKGTPDEVREKAKAFVEKIRGLTEGSLEHHHHHH
I53-50B.4PT1	MNQHSHKDHETVRIAVVRARWHAEIVDACVSAFEAAMRDIGGDRFAVDVFDVPGAYEIPLHARTLAETGRYGAVLGTAFVVNGGIYRHEFVASAVINGMMNVQLNTGVPVLSAVLTPHNYDKSKAHTLLFLALFAVKGMEAARACVEILAAREKIAA
2obx-wt	MNQHSHKDYETVRIAVVRARWHADIVDQCVSAFEAEMADIGGDRFAVDVFDVPGAYEIPLHARTLAETGRYGAVLGTAFVVNGGIYRHEFVASAVIDGMMNVQLSTGVPVLSAVLTPHNYHDSAEHHRFFFEHFTVKGKEAARACVEILAAREKIAAGSLEHHHHHH

* Mouse Igκ signal peptide and His tag sequences underlined.
